# Establishment of a Rapid *Escherichia coli* Detection Method Based on MIRA-*Pf*Ago

**DOI:** 10.3390/bios16050248

**Published:** 2026-04-29

**Authors:** Xinjun Chen, Yayun Liu, Jieru Wang, Yin Dai, Xuehuai Shen, Xiaocheng Pan, Dongdong Yin

**Affiliations:** 1 Yebio Bioengineering Co., Ltd. of Qingdao, Qingdao 266113, China; chenxinjun@yebio.com; 2 Anhui Provincial Key Laboratory of Livestock and Poultry Product Safety, Institute of Animal Husbandry and Veterinary Science, Anhui Academy of Agricultural Sciences, Livestock and Poultry Epidemic Diseases Research Center of Anhui Province, Hefei 230031, China; lyy1785@aaas.org.cn (Y.L.); wangjieru@aaas.org.cn (J.W.); dalin2080@aaas.org.cn (Y.D.); shenxh@aaas.org.cn (X.S.)

**Keywords:** *Escherichia coli*, multienzyme isothermal rapid amplification, *Pf*Ago, rapid nucleic acid detection

## Abstract

Conventional *Escherichia coli* (*E. coli*) detection methods are often time-consuming, while molecular diagnostics typically rely on expensive thermocycling equipment. To address these limitations, this study developed a rapid nucleic acid detection method for *E. coli* by integrating multienzyme isothermal rapid amplification (MIRA) with *Pyrococcus furiosus* Argonaute (*Pf*Ago)-mediated targeted cleavage. The conserved housekeeping gene *phoA* was selected as the target, and specific MIRA primers and 5′-phosphorylated guide DNAs (gDNAs) were designed accordingly. After exponential amplification at 39 °C, the amplicons were specifically recognized by *Pf*Ago at 95 °C, leading to molecular beacon cleavage and generation of a detectable FAM fluorescence signal. Among the tested guides, gDNA6 showed the highest cleavage efficiency. Optimal performance was achieved with 1 μM *Pf*Ago, 0.5 μM gDNA, and 5 mM MnCl_2_. The optimized MIRA-*Pf*Ago assay demonstrated a limit of detection of 10^0^ copies/μL, comparable to qPCR, and exhibited high specificity with no cross-reactivity against common enteric pathogens. In 28 clinical and environmental samples, the assay results were fully consistent with those of qPCR. Overall, the MIRA-*Pf*Ago platform provides a rapid, sensitive, and specific approach for *E. coli* detection, demonstrating strong potential to reduce reliance on precision thermal cyclers for resource-limited applications.

## 1. Introduction

*Escherichia coli* (*E. coli*) is one of the most prevalent Gram-negative bacteria inhabiting the intestinal tracts of humans and animals. As an established indicator of fecal contamination, it is widely used in water quality assessment, food safety surveillance, and environmental monitoring [1,2]. Certain pathogenic strains, such as enterohemorrhagic *E. coli* O157:H7, can cause severe foodborne illnesses and other clinical infections, posing a major threat to global public health [3,4]. Therefore, the development of rapid, sensitive, and specific methods for *E. coli* detection is of considerable importance for the timely control of foodborne outbreaks, clinical diagnosis, and environmental risk assessment.

Conventional detection of *E. coli* primarily relies on culture-based isolation followed by biochemical identification or serological typing. Although this approach is regarded as the gold standard, it is labor-intensive, time-consuming, and poorly suited for the detection of viable but non-culturable (VBNC) bacteria [5]. Molecular approaches, including quantitative real-time PCR (qPCR) and digital PCR, have substantially improved analytical sensitivity and specificity; however, their dependence on costly thermocycling instruments and well-equipped laboratory facilities limits their applicability in point-of-care testing and other resource-constrained settings [6,7].

In recent years, isothermal nucleic acid amplification technologies, particularly multienzyme isothermal rapid amplification (MIRA), have attracted increasing attention because of their rapid reaction kinetics, low instrumentation requirements, and elimination of thermal cycling. MIRA enables exponential amplification at a constant temperature of 20–41 °C through the coordinated action of recombinase, single-stranded DNA-binding proteins, and DNA polymerase [8,9,10]. Its sensitivity is comparable to that of conventional PCR, while the workflow is generally simpler and faster. Nevertheless, when applied to complex clinical or environmental specimens, standalone isothermal amplification methods may still be susceptible to false-positive results caused by nonspecific amplification. To address this limitation, secondary verification systems such as CRISPR/Cas systems have been widely explored; however, their application remains strictly constrained by the requirement of specific protospacer adjacent motif (PAM) sequences and a reliance on relatively unstable RNA guides. In contrast, *Pf*Ago (*Pyrococcus furiosus* Argonaute) is a DNA-guided endonuclease that exhibits highly efficient target cleavage activity at elevated temperatures [11,12,13]. Unlike CRISPR/Cas systems, the *Pf*Ago platform does not require PAM sequences, and its DNA oligonucleotide guides are inherently more stable, as well as easier to design and store [14]. By introducing *Pf*Ago-mediated secondary recognition of amplification products and subsequent molecular beacon cleavage, background interference can be effectively reduced, thereby providing a practical strategy for constructing a highly specific detection platform [15,16].

The *phoA* gene, which encodes alkaline phosphatase in *E. coli*, is a conserved housekeeping gene that is stably present in nearly all *E. coli* strains and has been validated in multiple studies as a reliable species-specific detection target [17,18]. Compared with other commonly used genetic markers, such as *uidA* and *gadAB*, *phoA* shows lower cross-reactivity with phylogenetically related members of the Enterobacteriaceae. With appropriately designed primers, this gene therefore enables accurate discrimination of *E. coli* from closely related bacteria.

In this study, we sought to combine the amplification efficiency of MIRA with the sequence discrimination capability of *Pf*Ago to establish a novel nucleic acid assay for *E. coli* detection. Using the *phoA* gene as the target, rapid amplification was first achieved by MIRA, followed by *Pf*Ago-based specific recognition and fluorescence signal generation. This strategy was designed to reduce dependence on precision thermocycling equipment while maintaining sensitivity and specificity comparable to those of qPCR, with a substantially shortened turnaround time. It is anticipated that this method will provide a molecular diagnostic tool with strong translational potential for food safety testing, environmental surveillance, and rapid clinical screening.

## 2. Materials and Methods

### 2.1. Bacterial Strains and Samples

The *E. coli* (ATCC 25922), *Salmonella pullorum* (ATCC 10398), *Salmonella typhimurium* (CICC 22956), *Shigella* (ATCC 12022), *Klebsiella pneumoniae* (ATCC 13883), *Listeria monocylogenes* (ATCC 19112), and *Staphylococcus aureus* (ATCC 29213), were all preserved in our laboratory. All strains were cultured in LB medium, after which genomic DNA was extracted and stored at −20 °C until use. A total of 28 animal clinical and environmental samples, including water, food, and animal fecal swab specimens, were collected from Anhui Province and surrounding areas.

### 2.2. Design of MIRA Primers and gDNAs

Specific primers for MIRA amplification were designed based on the *phoA* gene sequence of *E. coli* retrieved from GenBank. Six 5′-phosphorylated single-stranded DNA guide molecules (gDNAs) were designed to target internal regions of the MIRA amplicon. In addition, a sequence-specific molecular beacon labeled with a fluorescent reporter (FAM) at one end and a quencher (BHQ1) at the other end was designed for signal reporting (Table 1). All primers, gDNAs, and molecular beacon probes were synthesized by General Biosystems (Anhui), Co., Ltd. (Chuzhou, China).

### 2.3. Nucleic Acid Extraction and Preparation of the Standard Plasmid

Genomic DNA from bacterial strains and collected samples was extracted using a bacterial genomic DNA extraction kit (Tiangen Biotech Co., Ltd., Beijing, China). For environmental water and food samples, solid debris was first removed by low-speed centrifugation (500× *g* for 3 min), and the supernatant was concentrated before extraction. For fecal swabs, samples were thoroughly vortexed in sterile saline to release bacterial cells from the complex organic matrix prior to lysis. All extracted genomic DNA was stored at −20 °C until further use. To construct the standard plasmid, the target fragment of the *phoA* gene was cloned into the pUC57 vector, generating the recombinant plasmid pUC57-phoA. The recombinant plasmid was confirmed by sequencing, after which its concentration was determined and converted into copy number. The standard plasmid was then serially diluted 10-fold with nuclease-free water to concentrations ranging from 10^8^ to 10^0^ copies/μL and used as the template for subsequent sensitivity analysis.

### 2.4. Establishment of the MIRA-PfAgo Fluorescence Detection System

Isothermal rapid amplification was performed according to the instructions provided with the commercial amplification kit (Catalog No. WLB8201KIT, Amp-Future Biotech Co., Ltd., Weifang, China). The 25 μL MIRA reaction mixture consisted of 14.7 μL A Buffer, 1.0 μL each of forward and reverse primers (10 μM), 2.0 μL DNA template or standard plasmid, 1.25 μL B Buffer, and ddH_2_O added to a final volume of 25 μL. The reaction mixture was transferred into a tube containing lyophilized enzyme pellets (primarily recombinase, single-stranded DNA-binding proteins, and DNA polymerase) and incubated at 39 °C for 30 min. Subsequently, 2 μL of the MIRA amplification product was added to a 20 μL *Pf*Ago reaction system containing 150 mmol/L Tris-HCl (pH 8.0), 2.5 M NaCl, 5 mM MnCl_2_, 1 μM *Pf*Ago, 0.5 μM gDNA, and 2 μM molecular beacon, with ddH_2_O added to adjust the final volume to 20 μL. The reaction mixture was incubated at 95 °C for 30 min in an ABI 7500 real-time quantitative PCR instrument (Thermo Fisher Scientific, Waltham, MA, USA), and fluorescence signals in the FAM channel were recorded every 30 s.

### 2.5. Specificity Analysis

The optimized MIRA-*Pf*Ago assay was used to detect DNA extracted from *E. coli* and other common bacterial species. Nuclease-free water was included as the negative control (NTC). The method was considered to exhibit good specificity when a significant fluorescence signal was observed only in the *E. coli* sample, whereas no detectable fluorescence signal was generated from the other bacterial species or the negative control.

### 2.6. Sensitivity Analysis

Tenfold serial dilutions of the standard plasmid (10^6^–10^−1^ copies/μL) were used as templates for MIRA-*Pf*Ago fluorescence detection. In parallel, qPCR was performed for comparison according to a previously reported method [17]. Both assays were conducted simultaneously on the same batch of diluted standards. The analytical sensitivity of the MIRA-*Pf*Ago assay was evaluated by comparing the limit of detection (LOD) of the two methods.

### 2.7. Validation Using Clinical Samples

A total of 28 practical samples were tested using the established method. With qPCR serving as the reference standard, the diagnostic sensitivity, specificity, and overall concordance of the MIRA-*Pf*Ago assay were calculated to assess its reliability in practical applications.

### 2.8. Statistical Analysis

All experiments were performed in three independent technical replicates to ensure assay reliability, and the data are presented as the mean ± standard deviation (SD). Statistical analysis and data visualization were performed using GraphPad Prism software (version 8.0, GraphPad Software, San Diego, CA, USA). Differences between groups were analyzed using either an independent Student’s *t*-test or one-way analysis of variance (ANOVA). A *p*-value of <0.05 was considered to indicate a statistically significant difference (* *p* < 0.05, ** *p* < 0.01).

## 3. Results

### 3.1. Principle of the MIRA-PfAgo Detection Method

**Figure 1:**
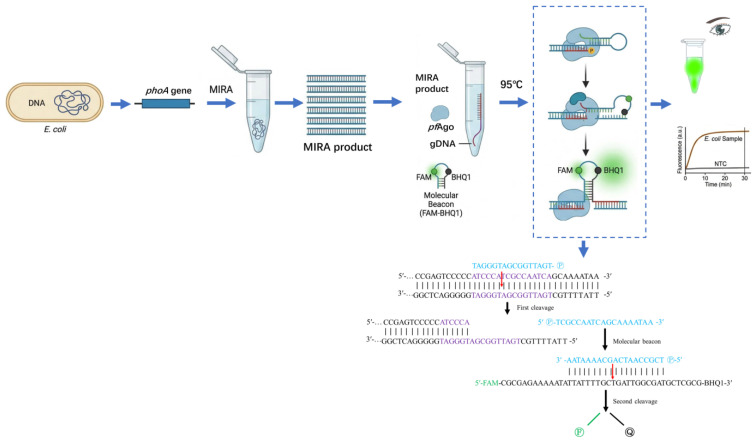
Schematic illustration of the MIRA-*Pf*Ago detection principle. The target *phoA* gene is exponentially amplified by MIRA at 39 °C, followed by *Pf*Ago-mediated specific cleavage at 95 °C under the guidance of 5′-phosphorylated gDNA. This specific cleavage event disrupts the stem-loop structure of the molecular beacon, separating the FAM fluorophore from the BHQ1 quencher and generating a detectable fluorescence signal.

The principle of the MIRA-*Pf*Ago detection system established in this study is illustrated in Figure 1. Briefly, the target *phoA* gene fragment was first exponentially enriched by MIRA amplification at 39 °C. The resulting amplicons were then specifically recognized and cleaved by *Pf*Ago under the guidance of 5′-phosphorylated gDNA at 95 °C. The gDNA guides *Pf*Ago to precisely bind and introduce a single-strand break within the target sequence. This cleavage process subsequently acts upon the added sequence-specific molecular beacon. Once the target region on the beacon is cleaved by the *Pf*Ago-gDNA complex, the stem-loop structure of the molecular beacon is destabilized and forced open, leading to the physical separation of the FAM fluorophore from the BHQ1 quencher. This separation eliminates the quenching effect, thereby generating a detectable fluorescence signal. By integrating the high amplification efficiency of MIRA with the precise target cleavage capability of *Pf*Ago, this method enables rapid detection of *E. coli* without the need for conventional thermal cycling.

### 3.2. Screening of gDNAs

**Figure 2:**
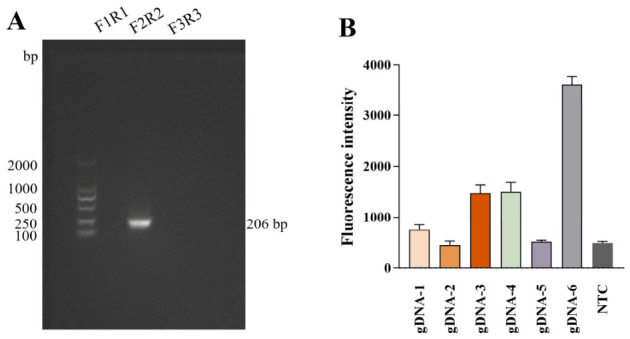
Screening of MIRA primers and gDNAs. (**A**) Gel electrophoresis of MIRA products using three candidate primer pairs (F1/R1, F2/R2, F3/R3). (**B**) Evaluation of six gDNAs based on endpoint fluorescence intensity. gDNA6 exhibited the highest cleavage efficiency. NTC: negative control.

Three pairs of primers were designed for screening MIRA primers targeting the *phoA* gene. As shown in Figure 2A, the primer pair F2/R2 amplified the expected target fragment with no nonspecific bands, yielding an amplicon of 206 bp. Therefore, this primer pair was selected for subsequent MIRA amplification of *E. coli*. Six gDNAs were designed to target internal regions of the MIRA amplicon. As shown in Figure 2B, gDNA6 produced the strongest fluorescence intensity, whereas the signals from other gDNAs were significantly weaker. Therefore, gDNA6 was selected as the optimal guide strand for all subsequent experiments.

### 3.3. Optimization of the MIRA-PfAgo Reaction Conditions

**Figure 3:**
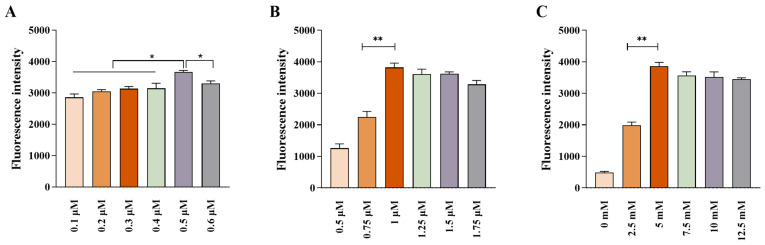
Optimization of the MIRA-PfAgo reaction conditions. Effects of (**A**) gDNA concentration (with fixed 1 μM *Pf*Ago and 5 mM MnCl_2_), (**B**) *Pf*Ago protein concentration (with fixed 0.5 μM gDNA and 5 mM MnCl_2_), and (**C**) MnCl_2_ concentration (with fixed 0.5 μM gDNA and 1 μM *Pf*Ago) on fluorescence intensity. Data are presented as the mean ± SD (n = 3). * *p* < 0.05, ** *p* < 0.01.

Key parameters of the *Pf*Ago reaction system were systematically optimized, including the concentrations of gDNA, *Pf*Ago protein, and MnCl_2_. The results showed that the fluorescence intensity reached a maximum when the gDNA concentration was 0.5 μM (Figure 3A). The strongest fluorescence signal was observed at a *Pf*Ago protein concentration of 1 μM (Figure 3B), while the highest fluorescence peak was obtained when the MnCl_2_ concentration was 5 mM (Figure 3C). Based on these findings, the optimal reaction conditions were determined to be 0.5 μM gDNA, 1 μM *Pf*Ago protein, and 5 mM MnCl_2_, and these parameters were applied in all subsequent experiments.

### 3.4. Specificity Analysis of the MIRA-PfAgo Assay

**Figure 4:**
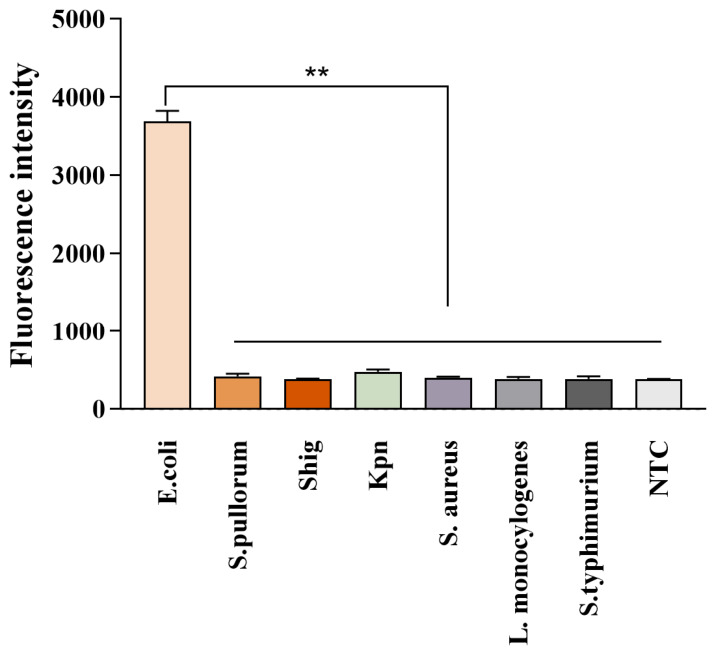
Specificity of the MIRA-*Pf*Ago assay. The system was tested against *E. coli* and six other common enteric bacteria. Significant fluorescence was exclusively observed for *E. coli*. NTC: negative control. Data are presented as the mean ± SD (n = 3). ** *p* < 0.01.

The optimized MIRA-*Pf*Ago system was applied to detect a panel of common enteric bacterial strains in order to evaluate its species specificity. As shown in Figure 4, only *E. coli* generated a strong FAM fluorescence signal, whereas no obvious fluorescence signal was detected from the DNA of other bacterial species or from the NTC. These results indicate that the established MIRA-*Pf*Ago assay possesses high specificity for *E. coli* and shows no cross-reactivity with common phylogenetically related enteric bacteria.

### 3.5. Sensitivity Analysis of the MIRA-PfAgo Assay

**Figure 5:**
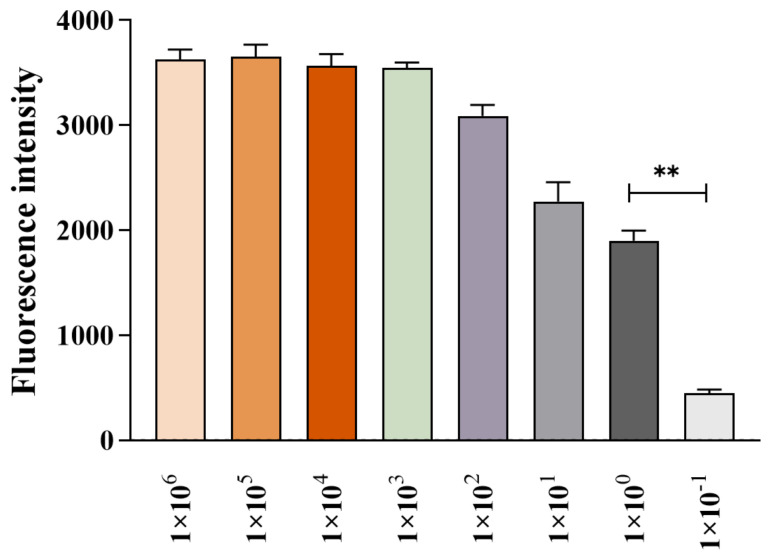
Sensitivity of the MIRA-*Pf*Ago assay. Tenfold serial dilutions of the standard *phoA* plasmid (10^6^ to 10^−1^ copies/μL) were tested to determine the LOD (10^0^ copies/μL). Data are presented as the mean ± SD (n = 3). ** *p* < 0.01.

Tenfold serial dilutions of the standard plasmid (10^6^–10^−1^ copies/μL) were used as templates to determine the detection limit of the established assay. The results showed that the MIRA-*Pf*Ago method achieved a limit of detection of 10^0^ copies/μL, at which a clear and detectable fluorescence signal was still observed, whereas no signal was detected at lower concentrations. The detection limit of qPCR was comparable to that of the proposed assay (Figure 5). These findings indicate that the MIRA-*Pf*Ago platform exhibits analytical sensitivity equivalent to that of qPCR and is suitable for detecting low-abundance *E. coli* samples.

### 3.6. Detection of Clinical Samples

**Figure 6:**
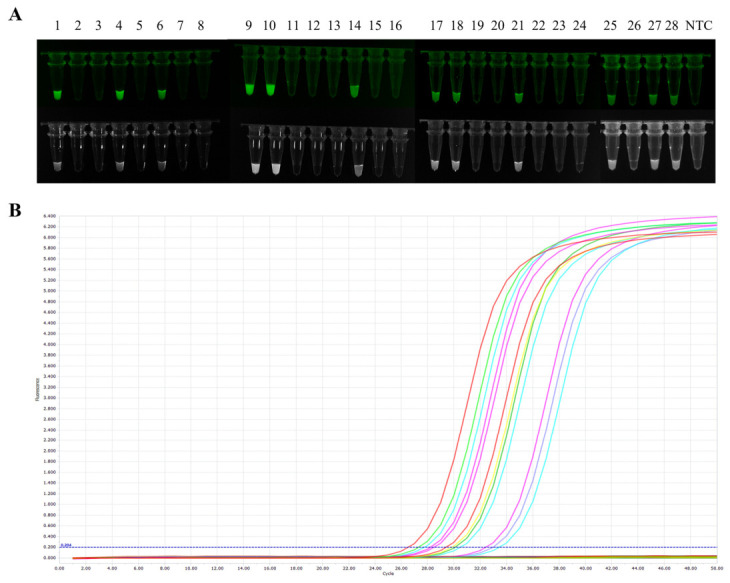
Clinical samples were detected by using MIRA-*Pf*Ago and qPCR. (**A**) Detection results of the MIRA-*Pf*Ago, identifying 12 positive samples and 16 negative samples. (**B**) The detection results of qPCR assay for 28 clinical samples. NTC: negative control.

A total of 28 clinical and environmental samples were tested using both the MIRA-*Pf*Ago assay established in this study and qPCR. The results showed that the MIRA-*Pf*Ago method identified 12 positive samples and 16 negative samples (Figure 6A). The positive percent agreement and negative percent agreement with qPCR were both 100% (Figure 6B). The calculated kappa value was 1.0, indicating that the MIRA-*Pf*Ago assay demonstrated excellent accuracy and strong reliability in the detection of practical samples.

## 4. Discussion

In this study, we successfully established a detection platform that integrates MIRA with *Pf*Ago-mediated target cleavage, using the conserved *E. coli* gene *phoA* as the detection target. Following 30 min of exponential amplification at 39 °C, the reaction products were directly subjected to the *Pf*Ago reaction at 95 °C, in which *Pf*Ago, guided by gDNA, specifically cleaved the amplicons and activated the molecular beacon to generate a FAM fluorescence signal. The entire workflow could be completed within 60 min without the need for precision thermocycling equipment. Compared with conventional CRISPR/Cas-based systems, *Pf*Ago offers notable advantages, including highly efficient target cleavage activity and a DNA-guided mechanism that is free from PAM sequence constraints, thereby substantially expanding the flexibility and applicability of target sequence design [19,20,21].

The *phoA* gene is highly conserved within *E. coli* species but shows greater sequence divergence among closely related members of the Enterobacteriaceae, making it a more suitable target than commonly used markers such as uidA or 16S rDNA [18]. Among the gDNAs evaluated in this study, gDNA6 exhibited the strongest cleavage activity and generated robust fluorescence signals in both the reference strain ATCC 25922 and clinical samples, while showing no cross-reactivity with *S. pullorum*, *S. aureus*, *L. monocylogenes*, or other tested bacterial species. These results confirm the high specificity of the proposed assay. It is worth noting that while the phoA gene allows excellent discrimination, environmental matrices often harbor complex microbial communities, including atypical strains and other Enterobacteriaceae such as *Shigella*. In our validation, closely related pathogens like *S. pullorum* and *Shigella* showed no cross-reactivity, indicating that the dual-recognition mechanism effectively prevents false positives caused by homologous sequences in related intestinal bacteria. To further highlight the advantages of this system, we benchmarked the MIRA-*Pf*Ago platform against other recently reported nucleic acid detection technologies (Table 2).

As demonstrated in Table 2, compared with CRISPR/Cas12a-based systems, the present assay relies on highly stable DNA guides and completely circumvents PAM sequence constraints, offering greater flexibility in target design. In comparison with LAMP, which relies on four to six complex primers and is therefore more prone to nonspecific amplification and false-positive signals, the present method requires only a single pair of MIRA primers. When coupled with *Pf*Ago-mediated secondary sequence verification, this design substantially improves interference resistance and sequence tolerance. Relative to MIRA or RPA alone, the incorporation of *Pf*Ago markedly enhances signal specificity [22,23]. The limit of detection of this method was consistently as low as 10^0^ copies/μL, which was comparable to that of qPCR. Moreover, complete agreement with qPCR was observed in the testing of clinical and environmental samples, further demonstrating the reliability of this approach in complex sample matrices.

In comparison with other reported *Pf*Ago-based platforms, the present method shows comparable reaction time and analytical sensitivity to previously described assays, including RPA-*Pf*Ago for methicillin-resistant *S. aureus* detection (55 min, LOD: 10^2^ copies/μL) [24], *Mycoplasma synoviae* detection (40 min, LOD: 2 copies/mL) [25], LAMP-*Pf*Ago for porcine epidemic diarrhea virus detection (35 min, LOD: 2.4 copies/μL) [26], and *Pf*Ago-based detection of Zika virus [27], while offering the additional advantage of simpler primer design. Although a LAMP-CRISPR/Cas12a assay for *E. coli* O157:H7 has also achieved visual detection, it remains constrained by PAM sequence requirements and the inherent risk of false-positive amplification [28,29]. In contrast, the *Pf*Ago-based system developed here effectively overcomes these limitations.

**Table 2:** Performance comparison of the MIRA-*Pf*Ago assay with existing molecular technologies.

Method	Target Pathogen	Isothermal/Thermal	Reaction Time	PAM Requirement	LOD
qPCR [17]	*E. coli*	Thermal	>90 min	No	10^1^ copies/μL
LAMP-CRISPR/Cas12a [28]	*E. coli* O157:H7	Isothermal	60 min	Yes	10^0^ CFU/mL
MIRA-*Pf*Ago	*E. coli*	Isothermal + 95 °C	60 min	No	10^0^ copies/μL

Although the MIRA-*Pf*Ago system showed complete agreement with qPCR across different matrices, testing only 28 samples is a clear constraint. Larger-scale studies will be needed to confirm how well the method handles various matrix inhibitors in the real world. Additionally, the platform is not yet ready for true point-of-care use in austere environments. The requirement for a 95 °C heating step, the reliance on fluorescence readers, and the current expense of *Pf*Ago enzymes all restrict immediate field deployment. Future efforts may draw on one-pot LAMP-CRISPR/Cas12a or RPA-CRISPR strategies to develop a closed-tube assay format that would completely eliminate aerosol contamination [30,31]. Furthermore, by expanding the gDNA set, the platform could be adapted for multiplex detection of pathogenic strains such as O157:H7 or for simultaneous screening of multiple pathogens, thereby providing a more translationally valuable tool for environmental surveillance and public health emergency response.

## 5. Conclusions

In this study, we established an MIRA-*Pf*Ago platform targeting the *phoA* gene for *E. coli* detection. Combining MIRA’s amplification efficiency with *Pf*Ago’s PAM-free precision, the platform achieved a sensitivity of 10^0^ copies/μL and strong specificity. It also achieved 100% diagnostic agreement with standard qPCR across 28 practical environmental and clinical samples. This dual-recognition workflow eliminates the need for thermal cycling and unstable RNA guides, offering a accurate, and rapid molecular diagnostic tool. With future optimizations for cost and portability, this platform holds great promise for food safety surveillance, environmental monitoring, and clinical diagnostics.

## Figures and Tables

**Table 1:** Sequences used in this study.

Reaction	Name	Sequence (5′–3′)
MIRA	*pho*A-F1	GTGCTCGCCGTTTAACGGGTGATCAGACTGC
	*pho*A-R1	GTATTGCCCGGTAAGCGGTAAGGCATCTATAC
	*pho*A-F2	GTGATCAGACTGCCGCTCTGCGTGATTCTC
	*pho*A-R2	CAGCGCATAGTGAGTGTATTGCCCGGTAAG
	*pho*A-F3	CTCTGCGTGATTCTCTTAGCGATAAACCTGC
	*pho*A-R3	GCAGCCGAGTCGGTGACGTAGTCCGGTTTGC
*Pf*Ago	gDNA1	P-TTTTGCTGATTGGCGA
	gDNA2	P-GCTGATTGGCGATGGG
	gDNA3	P-TATTTTGCTGATTGGC
	gDNA4	P-ATTGGCGATGGGATGG
	gDNA5	P-TTGCTGATTGGCGATG
	gDNA6	P-TGATTGGCGATGGGAT
	MB	FAM-cgcgagAAAAATATTATTTTGCTGATTGGCGATGctcgcg-BHQ1

## Data Availability

The original contributions presented in the study are included in the article; further inquiries can be directed to the corresponding authors.

## Abbreviations

The following abbreviations are used in this manuscript:

qPCR	quantitative real-time PCR
MIRA	multienzyme isothermal rapid amplification
PAM	protospacer adjacent motif
NTC	negative control
